# Neck Circumference and Incidence of Diabetes Mellitus over 10 Years in the Korean Genome and Epidemiology Study (KoGES)

**DOI:** 10.1038/srep18565

**Published:** 2015-12-18

**Authors:** Nam H. Cho, Tae Jung Oh, Kyoung Min Kim, Sung Hee Choi, Jae Ho Lee, Kyong Soo Park, Hak Chul Jang, Jong Yeol Kim, Hong Kyu Lee, Soo Lim

**Affiliations:** 1 Department of Preventive Medicine, Ajou University School of Medicine, Suwon, Korea; 2 Division of Endocrinology,Seoul National University College of Medicine and Seoul National University Bundang Hospital, Seongnam, Korea; 3 Division of Pulmonology, Seoul National University College of Medicine and Seoul National University Bundang Hospital, Seongnam, Korea; 4 Department of Internal Medicine, Seoul National University College of Medicine, Seoul, Korea; 5 Division of Constitutional Medicine and Diagnosis Research Group, Korea Institute of Oriental Medicine, Daejeon, Korea; 6 Department of Internal Medicine, Eulji University, Seoul, Korea

## Abstract

Neck circumference, a proxy for upper-body fat, may be a unique fat depot that indicates metabolic risk beyond whole body fat. We investigated whether neck circumference is associated with development of diabetes mellitus (DM) in a subset of data with Korean Genome and Epidemiology Study (n = 3521, age range = 42–71 years). Nondiabetic subjects at the baseline were categorized into 4 groups (Q1–Q4) according to their neck circumference. Parameters related with β-cell function and insulin resistance including Epworth sleepiness scale and snoring habit were examined. The development of DM was confirmed biannually based on a 75-g oral glucose tolerance test. Over the 10 years, 2623 (74.5%) among 3521 subjects were followed-up. Among them, 632 (24.1%) developed DM. The incidence of DM increased from 17.6% in Q1 to 18.2% in Q2, to 25.4% in Q3, and to 36.0% in Q4 (P < 0.001). After adjusting for most risk factors related with DM, the relative risks of DM development were 0.989 (95% confidence interval, 0.638–1.578), 1.660 (1.025–2.687), and 1.746 (1.037–2.942) in men and 0.939 (0.540–1.769), 1.518 (0.808–2.853), and 2.077 (1.068–4.038) in women in Q2, Q3, and Q4, respectively when compared to Q1. This finding indicates negative impact from large neck circumference in the development of DM.

Regional adipose tissue handles and stores excess dietary energy, which may have substantial cardiometabolic implications. Thus, distribution of this regional adipose tissue or ectopic fat may be an important predictor for cardiometabolic and vascular risks in addition to overall obesity. Among various ectopic fat deposition, the visceral adipose tissue (VAT) is regarded as the most pathogenic fat depot, indicating metabolic risk above and beyond the standard obesity indices^1^. It is well known that people with large amounts of visceral fat are at increased risk of insulin resistance, type 2 diabetes mellitus (T2DM), and cardiovascular disease (CVD)^2^,^3^,^4^. However, VAT does not account for all cardiometabolic risk. Recently, ectopic fat depots in other areas are reported to contribute to the development of CVD^1^.

Waist circumference has long been used as a measure of central adiposity and many studies have reported that it is strongly associated with cardiovascular and metabolic risk^5^,^6^. However, it comprises both visceral and subcutaneous fats despite a strong correlation with VAT^7^. Conversely, neck circumference is a phenotype of upper body fat depot and it may also affect the cardiometabolic system. Neck circumference has been shown to be correlated positively with insulin resistance and biochemical components of the metabolic syndrome^8^,^9^. In the Framingham Heart Study, study participants with large neck circumference had various cardiometabolic risk factors when compared to those with small neck circumference even after adjustment for VAT and body mass index (BMI)^8^. A large Brazilian population-based study showed that neck circumference was correlated with high triglycerides and fasting glucose levels, low high-density lipoprotein (HDL)-cholesterol levels, and insulin resistance index^9^.

Systemic free fatty acid concentrations are primarily determined by upper-body subcutaneous fat^10^. Although there is no investigational study that compares the amount of free fatty acids from neck subcutaneous fat with abdominal subcutaneous fat, the fat amount in the neck area can be substantial according to the result of a study that measured fat volume around the neck using a computed tomography (CT)^11^. Much evidence suggests that an increase of circulating free fatty acid levels is associated with insulin resistance and impaired glucose metabolism^12^. In addition, it was demonstrated that higher levels of upper-body subcutaneous fat were associated with higher low-density lipoprotein (LDL) and lower HDL-cholesterol concentrations^13^. Thus, neck circumference may be an independent correlate of metabolic risk factors, above and beyond BMI and waist circumference^14^,^15^.

In different context, several studies reported that larger neck circumference was an independent risk factor for sleep apnea syndrome, which might be associated with insulin resistance^16^,^17^. Neck circumference was also associated with snoring, which might increase metabolic risk^18^.

So far, few studies have investigated neck circumference and its association with T2DM in a prospective manner, particularly from Asian studies. In this study, therefore, we investigated the association between neck circumference and DM development in a large community-based cohort of Koreans.

## Materials and Methods

### Study Population

In 2001, the Korean Center for Disease Control and Prevention launched the Korean Genome and Epidemiologic Study (KoGES), which was based on two communities in South Korea: the Ansung cohort for a rural community and the Ansan cohort for an urban community. The KoGES is an ongoing prospective study that involves a biennial examination. Details of KoGES and the methods used have been described previously^19^. In brief, 10038 subjects aged 40–69 years were recruited to partake in this study (around 5000 from each community). Each cohort has its own specialized research topic: respiratory diseases in Ansan and endocrine diseases in Ansung. Neck circumference was measured in Ansan as an anthropometric index related with respiratory diseases.

Of the 5020 subjects in Ansan cohort, 4023 finished the second follow-up in 2003-2004. Among them, 583 (11.6%) individuals were previously diagnosed with DM, and neck circumference was not measured in 916 individuals. After excluding these people, 3521 subjects (1784 men and 1737 women), whose neck circumference was measured in 2003-2004, were enrolled in the present study and followed up for a 10-year period. Every two years, the incidence of DM was confirmed based on the World Health Organization criteria^20^, using a 75-g oral-glucose-tolerance test (OGTT).

All subjects participated in the study voluntarily, and informed consent was obtained in all cases. The study protocol was approved by the Ethics Committee of KoGES at the Korean National Institute of Health and the study was performed in accordance with the approved guidelines.

### Measurement of Anthropometric Parameters

The height and body weight were measured using standard methods in light clothes, and BMI was calculated (weight divided by height squared, kg/m^2^). For central obesity, waist circumference was measured at the midpoint between the lower limit of the ribcage and the iliac crest. The body fat (%) was examined by a tetrapolar bioelectrical impedance analysis (Inbody 3.0^®^, Inbody, Seoul, Korea). Smoking habit was classified into three categories: non-, ex-, and current. The alcohol consumption status was categorized into three: non-, ex-, and current. Exercise habit was divided into two categories: none or irregular (≤1/week) and regular (≥2/week). One episode of exercise was defined as exercising for at least 30 min.

### Neck Circumference Measurement

Participants were asked to stand erect with their head positioned in the Frankfort horizontal plane. The superior border of a tape measure was placed just below the laryngeal prominence and applied perpendicular to the long axis of the neck. Neck circumference was measured to the nearest 0.1 cm, using a tape measure.

### Measurement of Biochemical Parameters

After fasting for 12 h, the circulating levels of glucose, total cholesterol, triglyceride, and HDL-cholesterol were measured, using a Hitachi 747 chemistry analyzer (Hitachi Ltd, Tokyo, Japan). The LDL-cholesterol level (mg/dl) was calculated using the following formula: [total cholesterol (mg/dl) – HDL-cholesterol (mg/dl) – triglyceride (mg/dl)/5)]^21^. The glycosylated hemoglobin (HbA1c) level was determined by high-performance liquid chromatography (Variant II; BioRad Laboratories, Hercules, CA, USA). The plasma insulin concentrations were measured by radioimmunoassay (LINCO kit, St Charles, MO, USA). White blood cell (WBC) and hemoglobin were measured using an autoanalyzer (Sysmex, Kobe, Japan). Fasting levels of creatinine, as well as alanine and aspartate aminotransferases (ALT and AST, respectively) were measured, using a Hitachi 747 automated analyzer. Plasma renin activity (PRA) was measured by radioimmunoassay using Cobra r-counter (PACKARD, Meriden, CT, USA). The circulating concentration of high-sensitivity C-reactive protein (hsCRP) was measured by immunoradiometric assay (ADVIA 1650, Bayer Diagnostics, Tarrytown, NY, USA).

### Definition of Diabetes Mellitus and Evaluation of Insulin Resistance and Pancreatic β-Cell Function

In a 12-h fasting state, a 75-g oral-glucose-tolerance test (OGTT) was conducted. Fasting and postglucose load at 60-min and 2-h plasma glucose and insulin concentrations were measured: FPG, PG60, PG120 and FPI, PI60, PI120, respectively. DM was defined as ≥126 mg/dl in fasting glucose or ≥200 mg/dl in postload 2-h glucose concentrations after 75-g OGTT based on the WHO criteria^20^. Apart from patients previously diagnosed with DM, all subjects underwent a 2-h 75-g OGTT at each biannual follow-up visit. To evaluate insulin resistance, a homeostasis model assessment of insulin resistance (HOMA-IR) was calculated, using the following formula: [fasting plasma insulin (μIU/ml) × fasting plasma glucose (mg/dl)/405]^22^. The insulinogenic index (IGI), which is an estimate of early insulin secretion, was produced by dividing the increase in insulin during the first 60-min by the increase in glucose during the same period [60–0 min insulin (IU/ml)/60–0 min glucose (mg/ml)]^23^.

### Definition of Hypertension and Antihypertensive Medications

Blood pressure was recorded three times in the morning after the subjects had been in a relaxed state for at least 10 min, and a 5 min rest period was allowed between each measurement. Hypertension was defined based on the study by Joint National Committee 7: ≥ 140/90 mmHg^24^ or antihypertensive medication. Among the 389 study participants taking antihypertensive medications, detailed information about antihypertensive drugs could be obtained from 136 (35.0%). Antihypertensive drugs in participants were classified as angiotensin-converting enzyme inhibitors or angiotensin II receptor blockers (n = 86, 22.1%), β-blockers (n = 51, 13.1%), calcium channel blockers (n = 132, 33.9%), diuretics (n = 59, 15.2%), α-blockers (n = 1.3, 1.2%), and unknown (n = 136, 35.0%). Some people took dual or triple therapy.

### Assessment of snoring, witnessed sleep apnea, and Epworth sleepiness scale

Study participants completed interviewer-administered questionnaires, including questions on their sleep habit and snoring. To measure the general level of daytime sleepiness, we used the Epworth sleepiness scale (ESS)^25^. The ESS consists of eight questions about the subject’s likelihood of dozing off or falling asleep in a particular situation that is commonly encountered in daily life. Respondents use a four-point scale from 0 to 3 for each of the eight questions. Subjects with ≥11 scores were identified to have daytime sleepiness.

Snoring frequency was assessed using a five-point scale, which was used in the previous analysis^26^: never, infrequently, 1-3 nights/week, 4-5 nights/week, and ≥6 nights/week. Individuals were grouped into non-snorers, occasional snorers (snoring ≤3 nights/week or infrequently) and habitual snorers (snoring ≥4 nights/week). Snoring status was confirmed by a bed partner or a family member in a subset of participants who lived together more than 1 year. In order to validate the questionnaire, a subset of 200 participants in KoGES were queried two weeks after the initial test regarding their snoring habits, using the test-retest reliability of the snoring questionnaire. Agreement between the responses was good, with a κ-statistic value of 0.73. Sleep apnea was diagnosed when a bed partner or family member witnessed a subject with ceased respiration for at least 10 seconds.

### Statistical Analysis

All of the data were expressed as means with standard deviations (SD), or as n with %. The skewed values such as HOMA-IR and hsCRP were normalized by logarithmic transformation before all analyses. Correlations between the variables were analyzed using Pearson’s correlation. Categorical variables were compared among neck circumference quartiles using a χ^2^ test. Comparisons of the baseline variables with respect to quartiles of neck circumference were analyzed using ANOVA for continuous variables.

We mathematically calculated the hazard ratios for incident DM, using Cox proportional hazards models with potential confounding parameters: adjusted for age, BMI or waist circumference, family history of DM, anti-hypertensive medication, triglycerides, alanine aminotransferase, hsCRP, PRA, HbA1c, HOMA-IR and IGI. Daytime sleepiness by Epworth sleepiness scale and snoring habit were further adjusted. There was no significant multicollinearity among the risk factors included in the regression models (all variation inflation factors were less than 5). The analyses were performed using IBM SPSS Statistics for Windows version 20.0 (IBM Corp., Armonk, NY, USA). For all tests, *P* < 0.05 was considered statistically significant.

## Results

### Baseline characteristics

The mean age was slightly but significantly higher in women than in men (49.8 ± 7.1 years in men vs. 50.6 ± 7.6 years in women, *P* < 0.01). The mean BMI was not different between genders (24.4 ± 2.7 kg/m^2^ in men vs. 24.5 ± 3.0 kg/m^2^ in women, *P* > 0.05). The mean ± SD of neck circumference (ranges) was 37.6 ± 2.0 (31.8–45.3) cm in men and 32.9 ± 1.8 (23.0℃40.0) cm in women, which significantly was larger in men than in women by 4.7 cm. Current and ex-smokers were much greater in men than women. Liver function enzyme activities and serum creatinine levels were greater in men than women. The baseline HbA1c levels, HOMA-IR, and IGI were not different between genders.

Among all subjects, 179 (10.0%) men and 210 (12.1%) women had been taking anti-hypertensive medications. In lipid-lowering medications, 1.5% of men and 2.1% of women had been taking statin or other lipid-lowering agents on a regular basis. There were more occasional and habitual snorers in men than women, although daytime sleepiness, which was estimated by ≥11 of the ESS was not different.

The anthropometric and biochemical characteristics of subjects according to the gender-specific quartiles of neck circumference are shown in Table 1. The mean BMI, waist circumference, and percentage body fat increased with the larger quartiles of neck circumference. There were increasing trends in the HbA1c levels and the fasting glucose and postload 2-h glucose concentrations with respect to the higher categories. The fasting and postload 2-h insulin concentrations and HOMA-IR also had similar increasing trends. The total cholesterol, triglyceride, and LDL-cholesterol levels increased, whereas the HDL-cholesterol and PRA levels decreased with the quartiles of neck circumference.

In the correlation analysis, neck circumference was correlated with most factors related to obesity, glucose metabolism, and lipid parameters in both genders (Table 2). Blood pressures and hsCRP levels were also positively correlated with neck circumference. In both gender, there were modest but significant negative correlations of neck circumference with plasma renin activity: *r* = −0.057, *P* = 0.016 in men and *r* = −0.151, *P* < 0.001 in women, respectively.

### Follow-up

**Figure 1:**
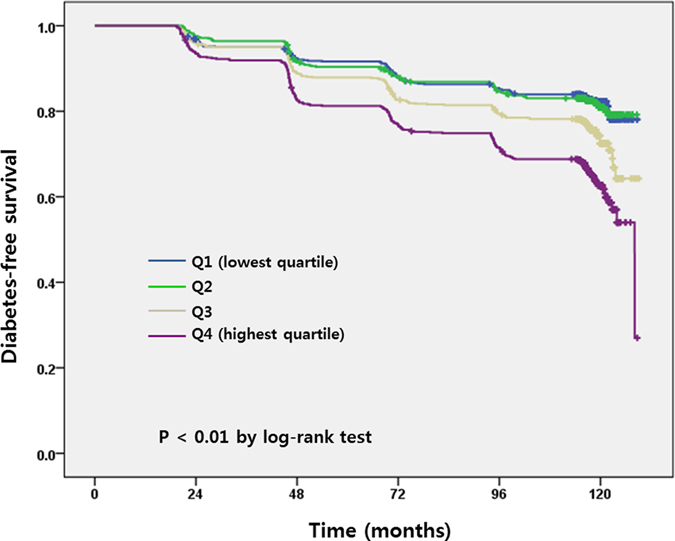
Diabetes mellitus-free survival curve in a 10-year period.

During the 10 year-study period, 2623 (74.5%) among 3521 subjects were followed-up. Of these subjects, 632 (24.1%) developed DM during the 10-year follow-up period. The mean ± SD of the follow-up duration was 104.9 ± 28.6 months (103.3 ± 29.9 months in men and 106.7 ± 27.2 months in women, respectively). The probability of developing DM increased in study subjects with higher quartiles of neck circumference compared to those with lowest quartile (*P* < 0.01) (Fig. 1).

Using the Cox proportional hazards models, we investigated the independent risk of neck circumference for the development of DM during the follow-up period by gender (Table 3). Factors that were significantly associated with DM incidence in univariate analysis or were known to be clinically important in the development of DM were selected as independent variables. Among the variables included in the final model, a high HbA1c concentration was the strongest predictive factor in the development of DM, regardless of gender. High HOMA-IR and low IGI were also significant factors. The relative risks (RRs) for the highest quartile of neck circumference were 1.746 in men and 2.077 in women (both *P* < 0.05). Older age, family history of DM, and high hsCRP levels were also associated with greater incidence of DM in both genders. High concentrations of triglycerides and ALT were also significant predictors in men. Of the antihypertensive agents, the uses of β-blockers in men and diuretics in women were associated with higher incidence of DM. Similar results were obtained with waist circumference instead of BMI: the RRs for the highest quartile of neck circumference were 1.575 (95% confidence interval (CI) 1.001–2.511; *P* = 0.048) in men and 2.062 (95% CI 1.050–4.050; *P* = 0.036) in women (Supplementary table 1). Waist circumference was also significantly associated with incidence of DM. Further adjustments for daytime sleepiness by Epworth sleepiness scale and snoring habit did not change the association.

Finally, when waist circumference was included in the final regression model instead of neck circumference, the highest quartile of waist circumference was independently associated with higher incidence of DM after adjusting for the same factors including BMI: 1.986 (95% CI 1.150–3.759; *P* = 0.035) in men and 2.045 (95% CI 1.000–4.551; *P* = 0.049) in women (Supplementary table 2).

## Discussion

In this prospective, community-based cohort study of Korean adults, we found that the highest quartile of neck circumference was associated with a 1.746 and 2.077 fold higher risk of DM development in men and women respectively, after adjusting for various factors that are known to affect glucose metabolism, including the HbA1c level, insulin resistance, β-cell function, liver enzyme activity, inflammation, as well as antihypertensive medications.

Previous studies provided a possibility of neck circumference as a cardiometabolic risk factor^8^,^9^,^27^,^28^. However, these were all cross-sectional studies and the study participants were not from population-based samples in some studies. A study using a population sample of 1,912 Turkish middle aged men and women showed that neck circumference was associated with metabolic syndrome more strongly than waist circumference^29^. In a recent study using Framingham Heart Study offspring participants, neck circumference was associated with increased carotid intima-media thickness but neither BMI nor waist circumference was associated^30^. These data support that neck circumference, a proxy of upper-body subcutaneous fat, may have a direct influence on atherosclerosis in adjacent vasculature. However, there was no study that investigated the role of neck circumference in the development of DM. In this context, our study has clinical importance of identifying an independent role of large neck circumference in the development of DM in a population-based large cohort.

Several mechanisms can be suggested underlying the association between large neck circumference and impaired glucose metabolism. Larger neck circumference alters peripheral blood flow and leads to endothelial function^31^, which may reduce insulin delivery and promote insulin resistance in the whole body^32^. A large study from Brazil showed a significant association between neck circumference and insulin resistance assessed using a euglycemic-hyperinsulinemic clamp^9^. In our study, neck circumference was positively correlated with triglycerides levels and negatively with HDL-cholesterol levels, both of which are robust markers for decreased insulin sensitivity^33^. Larger neck circumference with enhanced sympathetic activity may also contribute to insulin resistance, which may lead to the development of DM^34^.

In a different context, recent studies have reported that neck circumference is an independent predictor of nonalcoholic fatty liver disease, which is a strong indicator for T2DM^35^,^36^. Hepatic diacylglycerol content increases in the fatty liver, leading to, leading to activation of protein kinase Cϵ, which triggers impaired insulin signaling^37^.

Large neck circumference is structurally associated with pharyngeal narrowing and respiratory distress^38^. Repeated hypoxia and reoxygenation by airway obstruction—provoked by large neck circumference—may increase the production of reactive oxygen species, which also play an important role in the development of T2DM^39^.

Neck circumference was correlated with log-transformed hsCRP (*r* = 0.12, *P* < 0.001) and the hsCRP level was positively associated with an increased risk of DM in our study. *In vitro* and *in vivo* studies have shown that hsCRP and tumor necrosis factor-α (TNF-α), which are well-known inflammatory markers, play critical roles in the development of DM^40^,^41^. Thus, the systemic vascular resistance associated with large neck circumference accompanies oxidative stress and inflammation. Indeed, large neck circumference is associated with increased levels of cytokines, which are related to oxidative stress, such as TNF-α, interleukin-6, and nuclear factor *κ*-B, which in turn increases insulin resistance^42^. Taken together, large neck circumference might contribute to development of T2DM through various mechanisms.

Several studies have shown that neck circumference can be influenced by other factors. A study with retired National Football League players having calcium and plaque burden in the coronary artery showed that neck circumference was not associated with coronary or carotid subclinical atherosclerosis, which indicate that neck circumference may not be an appropriate marker for cardiometabolic risk^43^. Acute non-inflammatory status, such as cervical hematoma or vascular aneurysm, may increase the circumference of the neck^44^. Importantly, large neck circumference may be associated with lymph node metastasis in men with thyroid cancer^45^.

In this study, we adjusted for daytime sleepiness and snoring habit^25^,^26^. However, they were not associated with incidence of T2DM and did not change the association of neck circumference with T2DM. This result suggests that sleep habit may not have a robust role in the incidence of T2DM or it may be attenuated by other factors, such as insulin resistance or β-cell dysfunction.

The DM incidence rate of 27.7 per 1000 person-years in the current study seems to be slightly higher than that obtained from the 2009-2011 Korean National Data, showing 8-24 per 1000 person-years from a 40-69 year old population^46^. This may be because the current study was performed in early 2000s^46^.

Interestingly, we found in this study that high BMI was associated with higher incidence of DM in men but not in women. When waist circumference was used instead of BMI, larger waist circumference was significantly associated with higher incidence of DM. These results suggest that waist circumference may be a better indicator of insulin resistance than BMI.

The present study has several advantages. First, possible factors that may affect glucose regulation, such as age, BMI, lipids, liver function, PRA, hsCRP, antihypertensive drugs, and the HbA1c level were all adjusted. Daytime sleepiness and snoring status were also evaluated. Second, study participants were from a well-designed community-based cohort with a single ethnic group, who were within the 42–71 age group^47^. Third, dynamic indices for insulin resistance and β-cell function, which are not easily captured in clinical practice, were used in the regression model.

There are several limitations to be considered in this study. Detailed information about antihypertensive drugs could be obtained from only about 65% of the study participants. Information regarding changes and compliance in medications was not evaluated. Other variables that may be related to DM, such as apolipoprotein-B, lipoprotein (a), sex hormone binding globulin, gamma-glutamyl transpeptidase, or uric acid levels, were not measured. The neck circumference was not measured in the follow-up studies with this cohort.

In conclusion, to the best of our knowledge, this is the first longitudinal cohort study that reports the neck circumference as a predictive risk factor for future DM development in an Asian population. Neck circumference is a novel, easily measured fat depot, which may be an important predictor of DM. This fat depot may lead to a better understanding of systemic effect of ectopic fact on glucose homeostasis. This study provides a new insight into the underlying metabolic pathway between large neck circumference and DM. Future prospective studies are needed to better understand the extent to which a reduction of neck circumference may have in decreasing DM development.

## Additional Information

**How to cite this article**: Cho, N. H. *et al.* Neck Circumference and Incidence of Diabetes Mellitus over 10 Years in the Korean Genome and Epidemiology Study (KoGES). *Sci. Rep.* **5**, 18565; doi: 10.1038/srep18565 (2015).

## Supplementary Material

Supplementary Tables

## Figures and Tables

**Table 1:** Anthropometric and biochemical parameters in accordance to quartiles of neck circumference by gender.

	Men								Post hoc*	Women								Post hoc*
	Q1		Q2		Q3		Q4			Q1		Q2		Q3		Q4		
Neck circ. (cm)	35.1	0.9	37.0	0.4	38.4	0.4	40.3	1.1	a,b,c,d,e,f	30.7	0.8	32.2	0.3	33.5	0.3	35.2	0.4	b,c,e
(ranges)	(31.8–36.2)		(36.3–37.6)		(37.7–39.0)		(39.1–45.3)			(23.0–31.6)		(31.7–32.8)		(32.9–34.0)		(34.1–40.0)		
Age (years)	50.4	7.5	49.7	6.9	49.8	7.4	49.4	6.5	NS	49.3	7.0	50.1	7.5	51.2	7.6	51.9	8.2	b,c
SBP (mmHg)	110.0	14.5	111.9	13.4	114.6	15.5	115.8	13.7	b,c,d,e	105.7	14.1	107.3	15.9	110.4	15.3	114.8	15.8	b,c,d,e
DBP (mmHg)	73.0	10.1	74.7	9.6	77.0	10.8	78.0	10.4	a,b,c,d,e	68.3	9.7	69.5	10.3	71.3	10.4	74.2	10.5	b,c,d,e,f
BMI (kg/m^2^)	21.8	1.9	23.7	1.6	25.2	1.6	27.2	2.0	a,b,c,d,e,f	21.8	1.9	23.6	1.9	25.4	2.0	27.4	2.8	a,b,c,d,e,f
Waist circ. (cm)	76.3	6.0	81.3	4.8	85.0	4.7	89.6	5.4	a,b,c,d,e,f	70.0	5.1	74.3	5.3	78.3	5.1	83.3	6.4	a,b,c,d,e,f
Body fat (%)	18.1	4.4	20.2	4.1	22.4	3.7	24.7	4.0	a,b,c,d,e,f	27.7	4.6	30.2	4.3	32.1	4.4	34.3	4.4	a,b,c,d,e,f
WBC (×10^3^/μl)	5.9	1.7	6.1	1.7	6.4	1.7	6.6	1.7	b,c,d,e	5.2	1.5	5.5	1.4	5.6	1.5	6.2	1.5	b,c,e,f
Hb (g/dl)	14.6	1.0	14.7	1.0	14.9	1.0	15.1	0.9	b,c,d,e,f	12.4	1.1	12.4	1.1	12.5	1.1	12.7	1.2	c,e
AST (IU/l)	24.9	21.5	25.0	20.3	25.4	10.6	26.7	14.6	NS	20.4	5.9	20.5	6.5	21.0	6.4	22.1	10.7	c,e,f
ALT (IU/l)	23.4	18.4	25.6	30.3	28.2	16.2	32.8	19.6	b,c,e,f	16.2	7.0	17.2	8.6	19.1	10.4	21.6	15.0	b,c,d,e,f
Cr (mg/dl)	1.08	0.12	1.10	0.24	1.11	0.21	1.14	0.14	b,c,e	0.88	0.11	0.88	0.16	0.89	0.09	0.91	0.11	b,c,e
HbA1c (%)	5.31	0.37	5.32	0.34	5.35	0.35	5.42	0.41	c,e,f	5.23	0.35	5.29	0.36	5.31	0.35	5.47	0.40	a,b,c,e,f
FPG (mg/dl)	90.6	8.8	92.7	11.8	94.0	11.3	95.3	11.6	b,c,e	87.2	9.8	87.5	7.6	88.3	10.4	90.4	9.9	c,e,f
PG60 (mg/dl)	166.7	45.9	163.7	46.5	174.9	46.0	175.1	42.1	b,c	144.9	39.4	148.7	38.7	155.7	40.0	171.5	39.6	b,c,e,f
PG120 (mg/dl)	132.1	38.8	133.1	41.2	141.5	36.6	147.3	38.2	b,c,d,e	129.1	32.7	137.8	33.2	139.5	33.3	158.5	39.7	a,b,c,e,f
FPI (IU/ml)	7.5	2.6	8.3	4.3	8.9	3.1	10.2	3.7	a,b,c,e,f	8.3	2.8	9.0	4.5	9.0	3.1	10.6	4.0	c,e,f
PI60 (IU/ml)	35.1	26.3	40.6	33.2	45.1	32.5	50.4	42.4	a,b,c	36.4	27.4	44.6	35.1	44.5	31.5	58.3	45.3	c,e,f
PI120 (IU/ml)	30.1	26.1	33.5	28.3	41.7	37.5	44.6	36.0	b,c,d,e	39.4	28.7	48.2	37.4	51.9	45.3	70.7	55.8	b,c,e,f
HOMA-IR^†^	1.71	0.65	1.93	1.02	2.11	0.84	2.40	0.92	a,b,c,e,f	1.80	0.67	1.97	1.07	2.01	0.77	2.43	1.06	c,e,f
IGI	0.62	2.26	0.64	1.51	0.46	0.69	0.68	1.75	NS	0.74	2.42	0.79	1.36	0.76	1.22	0.60	1.19	NS
Total C (mg/dl)	191.8	31.4	196.0	29.6	201.1	32.8	206.1	34.0	b,c,d,e,f	195.5	34.9	202.5	33.6	206.8	36.8	209.8	36.0	a,b,c,e
TG (mg/dl)	126.6	99.4	134.5	78.0	161.5	98.9	188.9	120.2	b,c,d,e,f	97.0	62.4	108.6	62.9	119.3	65.7	144.6	73.8	a,b,c,d,e,f
HDL-C (mg/dl)	48.3	10.9	45.7	9.1	44.0	8.3	42.5	8.1	a,b,c,d,e,f	51.2	9.9	49.8	9.9	47.8	9.3	45.8	9.5	b,c,d,e,f
LDL-C (mg/dl)	120.8	30.7	124.6	28.7	127.5	30.3	132.4	35.7	b,c,e,f	125.5	31.3	131.7	30.3	135.9	32.0	136.1	32.6	a,b,c
PRA (ng/ml/h)^†^	3.2	2.6	3.2	2.9	3.0	2.5	3.1	3.2	NS	2.3	1.8	2.2	1.9	2.0	2.1	1.8	1.9	c,e
hsCRP (mg/l)^†^	1.02	2.06	0.96	1.53	1.37	2.06	1.54	2.32	c,e	1.12	3.46	0.89	1.60	1.03	1.42	1.72	2.34	c,e,f
ESS	5.6	3.3	6.1	3.6	6.0	3.8	6.3	3.9	c	6.6	4.1	6.2	3.7	6.3	3.9	6.1	3.9	NS

*Mean with SD. ^†^log-transformed values were used for statistical comparison. Abbreviation: WBC, white blood cell; Hb, hemoglobin; AST, aspartate aminotransferase; ALT, alanine aminotransferase; FPG, fasting plasma glucose; PG, postload glucose; FPI, fasting plasma insulin; PI, postload insulin; IGI, insulinogenic index; C, cholesterol; TG, triglyceride; PRA, plasma renin activity; hsCRP, high sensitivity C-reactive protein; ESS, Epworth sleepiness scale. *Post hoc analysis by Tukey’s-b *t* tests for mean differences between two groups: a, Q1 vs. Q2; b, Q1 vs. Q3; c, Q1 vs. Q4; d, Q2 vs. Q3; e, Q2 vs. Q4; f, Q3 vs. Q4, P < 0.05 in all cases; NS, not significant.

**Table 2:** Simple correlation of neck circumference with various parameters.

	Men		Women	
	*r*	P	*r*	P
Age (years)	−0.056*	0.018	0.139**	<0.001
SBP (mmHg)	0.170**	<0.001	0.203**	<0.001
DBP (mmHg)	0.200**	<0.001	0.199**	<0.001
BMI (kg/m^2^)	0.801**	<0.001	0.744**	<0.001
Waist circumference (cm)	0.740**	<0.001	0.706**	<0.001
Body fat (%)	0.547**	<0.001	0.510**	<0.001
WBC (×10^3^/μl)	0.163**	<0.001	0.229**	<0.001
AST (IU/l)	0.033	0.162	0.091**	<0.001
ALT (IU/l)	0.162**	<0.001	0.200**	<0.001
Creatinine (mg/dl)	0.120**	<0.001	0.090**	<0.001
HbA1c (%)	0.151**	<0.001	0.216**	<0.001
FPG (mg/dl)	0.159**	<0.001	0.122**	<0.001
PG60 (mg/dl)	0.087**	0.005	0.225**	<0.001
PG120 (mg/dl)	0.149**	<0.001	0.250**	<0.001
FPI (IU/ml)	0.283**	<0.001	0.206**	<0.001
PI60 (IU/ml)	0.165**	<0.001	0.232**	<0.001
PI120 (IU/ml)	0.184**	<0.001	0.245**	<0.001
HOMA-IR^†^	0.317**	<0.001	0.234**	<0.001
IGI^†^	0.070*	0.027	0.055	0.161
TG (mg/dl)	0.240**	<0.001	0.256**	<0.001
HDL-C (mg/dl)	−0.246**	<0.001	−0.223**	<0.001
PRA (ng/ml/h)^†^	−0.057*	0.016	−0.151**	<0.001
hsCRP (mg/l)^†^	0.114**	<0.001	0.091*	0.017
ESS	0.071**	0.003	−0.034	0.161

^*^P < 0.05, ^**^P < 0.01, ^†^log-transformed values were used for statistical comparison WBC, white blood cell; AST, aspartate aminotransferase; ALT, alanine aminotransferase; FPG, fasting plasma glucose; PG, postload glucose; FPI, fasting plasma insulin; PI, postload insulin; IGI, insulinogenic index; C, cholesterol; TG, triglyceride; PRA, plasma renin activity; hsCRP, high sensitivity C-reactive protein; ESS, Epworth sleepiness scale.

**Table 3:** Cox Proportional Hazards Model for Multiple Parameters to Assess the Association Between Neck Circumference and Incidence of Diabetes Mellitus by Gender.

	Men				Women			
	*P*	RR	95.0% CI Lower	95.0% CI Upper	*P*	RR	95.0% CI Lower	95.0% CI Upper
Age (years)	0.009	1.027	1.007	1.047	0.010	1.034	1.008	1.061
BMI (kg/m^2^)								
23.0-24.9 vs. <23.0	0.544	0.875	0.569	1.347	0.437	1.243	0.718	2.150
25.0-29.9 vs. <23.0	0.454	0.833	0.516	1.344	0.474	0.798	0.431	1.479
≥30.0 vs. <23.0	0.049	1.972	1.001	4.349	0.860	0.926	0.395	2.170
Family history of DM								
Yes vs. no	0.002	1.779	1.236	2.560	0.028	1.639	1.054	2.550
HT medications								
ACE inhibitors or ARBs	0.216	1.644	0.748	3.615	0.098	0.327	0.077	1.398
β-blockers	0.006	2.989	1.377	6.489	0.183	1.773	0.763	4.119
Calcium channel blockers	0.234	0.659	0.332	1.309	0.399	0.679	0.276	1.670
Diuretics	0.135	2.170	0.786	5.994	0.047	2.893	1.015	8.250
Others or unknown	0.364	1.259	0.765	2.072	0.764	1.102	0.584	2.082
Triglycerides (mg/dl)	0.005	1.002	1.001	1.003	0.682	1.001	0.998	1.003
ALT (mg/dl)	0.036	1.006	1.000	1.012	0.081	1.011	0.999	1.024
hsCRP (mg/l)	0.025	1.049	1.006	1.094	0.037	1.079	1.005	1.160
PRA (ng/ml/h)	0.782	0.995	0.959	1.032	0.876	1.006	.936	1.081
HbA1c (%)	<0.001	3.573	2.568	4.972	<0.001	4.641	2.739	7.863
Log(HOMA-IR)	<0.001	2.478	1.695	3.622	0.002	2.060	1.294	3.280
Insulinogenic index	<0.001	0.656	0.559	0.770	0.012	0.859	0.763	0.967
Neck circumference								
2^nd^ quartile vs. 1^st^ quartile	0.989	1.003	0.638	1.578	0.939	0.977	0.540	1.769
3^rd^ quartile vs. 1^st^ quartile	0.039	1.660	1.025	2.687	0.195	1.518	0.808	2.853
4^th^ quartile vs. 1^st^ quartile	0.036	1.746	1.037	2.942	0.031	2.077	1.068	4.038

ALT, alanine aminotransferase; hsCRP, high sensitivity C-reactive protein; PRA, plasma renin activity.
