# Supplementary material for: Neck Circumference and Incidence of Diabetes Mellitus over 10 Years in the Korean Genome and Epidemiology Study (KoGES)
Source: Sci Rep. 2015 Dec 18;5:18565. doi: 10.1038/srep18565 (PMC4683519; doi:10.1038/srep18565)
Supplement: Supplementary Tables [file srep18565-s1.doc]

**Supplementary tables**

**Title: Neck Circumference and Incidence of Diabetes Mellitus over 10 Years in the Korean Genome and Epidemiology Study (KoGES)**

Authors: Nam H. Cho^1^, Tae Jung Oh^2^, Kyoung Min Kim^2^, Sung Hee Choi^2^, Jae Ho Lee^3^, Kyong Soo Park^4^, Hak Chul Jang^2^, Jong Yeol Kim^5^, Hong Kyu Lee^6^ and Soo Lim^2^

^1^Department of Preventive Medicine, Ajou University School of Medicine, Suwon, Korea

^2^Division of Endocrinology, ^3^Division of Pulmonology, Seoul National University College of Medicine and Seoul National University Bundang Hospital, Seongnam, Korea

^4^Department of Internal Medicine, Seoul National University College of Medicine, Seoul, Korea

^5^Division of Constitutional Medicine and Diagnosis Research Group, Korea Institute of Oriental Medicine, Daejeon, Korea

^6^Department of Internal Medicine, Eulji University, Seoul, Korea

| **Supplementary Table 1. Cox Proportional Hazards Model for Multiple Parameters including waist circumference to Assess the Association Between Neck Circumference and Incidence of Diabetes Mellitus by Gender** | | | | | | | | |
| --- | --- | --- | --- | --- | --- | --- | --- | --- |
|  | Men | | | | Women | | | |
|  | *P* | RR | 95% CI  Lower | 95% CI  Upper | *P* | RR | 95% CI  Lower | 95% CI  Upper |
| Age (years) | 0.038 | 1.021 | 1.001 | 1.041 | 0.004 | 1.040 | 1.013 | 1.068 |
| Waist circumference (cm) | 0.021 | 1.025 | 1.018 | 1.036 | 0.032 | 1.021 | 1.011 | 1.039 |
| *Family history of DM* |  |  |  |  |  |  |  |  |
| Yes vs. no | 0.004 | 1.709 | 1.191 | 2.454 | 0.028 | 1.644 | 1.055 | 2.559 |
| *HT medications* |  |  |  |  |  |  |  |  |
| ACE inhibitor or ARBs | 0.184 | 1.655 | 0.787 | 3.479 | 0.152 | 0.349 | 0.082 | 1.476 |
| β-blockers | 0.007 | 2.921 | 1.349 | 6.324 | 0.253 | 1.642 | 0.702 | 3.843 |
| Calcium channel blockers | 0.212 | 0.646 | 0.326 | 1.283 | 0.410 | 0.687 | 0.282 | 1.676 |
| Diuretics | 0.142 | 2.147 | 0.774 | 5.957 | 0.028 | 3.206 | 1.137 | 9.042 |
| Others or unknown | 0.299 | 1.301 | 0.792 | 2.139 | 0.658 | 1.151 | 0.618 | 2.144 |
| Triglyceride (mg/dl) | 0.006 | 1.002 | 1.001 | 1.003 | 0.521 | 1.001 | 0.998 | 1.003 |
| ALT (mg/dl) | 0.027 | 1.007 | 1.001 | 1.012 | 0.074 | 1.011 | 0.999 | 1.024 |
| hsCRP (mg/l) | 0.022 | 1.050 | 1.007 | 1.095 | 0.018 | 1.088 | 1.014 | 1.166 |
| PRA (ng/ml/h) | 0.880 | 0.997 | 0.962 | 1.034 | 0.882 | 1.005 | 0.937 | 1.079 |
| HbA1c (%) | <0.001 | 3.517 | 2.531 | 4.887 | <0.001 | 4.537 | 2.685 | 7.666 |
| Log(HOMA-IR) | <0.001 | 2.359 | 1.609 | 3.457 | 0.002 | 2.104 | 1.315 | 3.367 |
| Insulinogenic index | <0.001 | 0.665 | 0.566 | 0.781 | 0.018 | 0.870 | 0.776 | 0.976 |
| *Neck circumference* |  |  |  |  |  |  |  |  |
| 2^nd^ vs. 1^st^ quartile | 0.620 | 0.895 | 0.579 | 1.385 | 0.883 | 1.045 | 0.582 | 1.874 |
| 3^rd^ vs. 1^st^ quartile | 0.109 | 1.427 | 0.924 | 2.205 | 0.242 | 1.447 | 0.780 | 2.684 |
| 4^th^ vs. 1^st^ quartile | 0.048 | 1.575 | 1.001 | 2.511 | 0.036 | 2.062 | 1.050 | 4.050 |

| **Supplementary Table 2. Cox Proportional Hazards Model for Multiple Parameters including BMI to Assess the Association Between Waist Circumference and Incidence of Diabetes Mellitus by Gender** | | | | | | | | |
| --- | --- | --- | --- | --- | --- | --- | --- | --- |
|  | Men | | | | Women | | | |
|  | *P* | RR | 95% CI  Lower | 95% CI  Upper | *P* | RR | 95% CI  Lower | 95% CI  Upper |
| Age (years) | 0.027 | 1.022 | 1.003 | 1.043 | 0.063 | 1.025 | 0.999 | 1.051 |
| BMI (kg/m^2^) |  |  |  |  |  |  |  |  |
| 23.0-24.9 vs. <23.0 | 0.466 | 0.841 | 0.529 | 1.338 | 0.805 | 1.074 | 0.607 | 1.902 |
| 25.0-29.9 vs. <23.0 | 0.636 | 0.881 | 0.522 | 1.487 | 0.406 | 0.755 | 0.388 | 1.467 |
| ≥30.0 vs. <23.0 | 0.098 | 1.982 | 0.873 | 4.502 | 0.957 | 1.025 | 0.418 | 2.513 |
| *Family history of DM* |  |  |  |  |  |  |  |  |
| Yes vs. no | 0.003 | 1.740 | 1.207 | 2.510 | 0.028 | 1.636 | 1.056 | 2.533 |
| *HT medications* |  |  |  |  |  |  |  |  |
| ACE inhibitors or ARBs | 0.157 | 1.754 | 0.806 | 3.818 | 0.178 | 0.367 | 0.085 | 1.578 |
| β-blockers | 0.007 | 2.902 | 1.338 | 6.294 | 0.092 | 2.078 | 0.886 | 4.870 |
| Calcium channel blockers | 0.379 | 0.735 | 0.370 | 1.459 | 0.668 | 0.825 | 0.343 | 1.985 |
| Diuretics | 0.027 | 3.153 | 1.137 | 8.744 | 0.021 | 3.375 | 1.205 | 9.452 |
| Others or unknown | 0.404 | 1.234 | 0.753 | 2.022 | 0.253 | 1.428 | 0.775 | 2.629 |
| Triglyceride (mg/dl) | 0.009 | 1.002 | 1.000 | 1.003 | 0.689 | 1.000 | 0.998 | 1.003 |
| ALT (mg/dl) | 0.037 | 1.006 | 1.000 | 1.012 | 0.043 | 1.013 | 1.000 | 1.026 |
| hsCRP (mg/l) | 0.016 | 1.054 | 1.010 | 1.099 | 0.039 | 1.081 | 1.004 | 1.164 |
| PRA (ng/ml/h) | 0.922 | 0.998 | 0.959 | 1.038 | 0.989 | 0.999 | 0.929 | 1.075 |
| HbA1c (%) | <0.001 | 3.469 | 2.496 | 4.823 | <0.001 | 4.665 | 2.762 | 7.878 |
| Log(HOMA-IR) | <0.001 | 2.223 | 1.525 | 3.239 | 0.002 | 2.138 | 1.336 | 3.422 |
| Insulinogenic index | <0.001 | 0.667 | 0.568 | 0.783 | 0.017 | 0.863 | 0.765 | 0.974 |
| *Waist circumference* |  |  |  |  |  |  |  |  |
| 2^nd^ vs. 1^st^ quartile | 0.198 | 1.405 | 0.837 | 2.357 | 0.265 | 1.466 | 0.748 | 2.874 |
| 3^rd^ vs. 1^st^ quartile | 0.486 | 1.234 | 0.683 | 2.227 | 0.042 | 1.980 | 1.001 | 4.063 |
| 4^th^ vs. 1^st^ quartile | 0.035 | 1.986 | 1.050 | 3.759 | 0.049 | 2.045 | 1.000 | 4.551 |
